# Determination of the Prevalence of *Ascaris lumbricoides* in Children under the Age of Five Years Attending at Kongowe Health Centre, Kibaha District, Pwani Region

**DOI:** 10.1155/2024/1932633

**Published:** 2024-05-15

**Authors:** Adnan A. Hamad, Godfrey O. Mauti

**Affiliations:** ^1^Department of Medical Laboratory Science, Faculty of Medicine and Pharmaceutical Sciences, Kampala International University in Tanzania, Dar es Salaam, Tanzania; ^2^Department of Physical and Biological Sciences, School of Pure and Applied Science, Bomet University College, Bomet, Kenya; ^3^Department of School of Allied Health Science, Kampala International University-Western Campus, Bushenyi, Uganda

## Abstract

**Background:**

The worldwide distribution and occurrence show that more than 1.8 billion people, accounting for 28% of the global population, are infected with *A. lumbricoides* infection due to poor hygiene. The World Health Organization aims to eliminate morbidity from *A. lumbricoides* infection in children by 2030 by at least 75%. Surveys in the Pwani Region of Tanzania have shown poor hygienic risk factors due to high population and lack of sufficient toilets/latrines, poor sewage waste disposal, and insufficient clean drinking water that encourages the reproduction, transmission, and reinfection of *A. lumbricoides.* No study has been conducted in Pwani Region to determine the extent of *A. lumbricoides* infection among the population. This research tends to explore more about the status of *A. lumbricoides* infection in Kibaha District in the Pwani Region, Tanzania.

**Methods:**

A cross-sectional study was done on 400 children (170 males and 230 females) who were under the age of 5 years old at Kongowe Health Centre. Collected data for *A. lumbricoides* infection measured were analyzed using Microsoft Excel, while data for sociodemographic findings and risk assessment were subjected to bivariate and multivariable analysis using Statistical Package for Social Sciences version 28.0.1.0 software (SPSS Inc., USA) at the level of significance of *P* < 0.05.

**Results:**

Of the 400 children who participated in the laboratory testing of *A. lumbricoides* infection, 194 were found positive for *A. lumbricoides* infection. Amongst the 194 children who were found to be positive, 4.1%, 38.7%, and 57.2% were found to be in the age group of below 1 year old, 1 to 2, and 3 to 4 years old, respectively. The tabulated responses on the social demographic responses showed that 81.8% of the responses at *P* = 0.041 and 80.6% at *P* = 0.101 knew that *A. lumbricoides* infection is acquired from faeces and uncooked food, respectively, as 61.8% at *P* = 0.132 and 63.2% at *P* = 0.085 respondents did not accept that soil and dirty water, respectively, were not sources of *A. lumbricoides* infection. At *P* = 0.612 and *P* = 0.022, 64% and 91.2% of the respondents showed the importance of using latrines/toilets and washing fruits, respectively. Further results showed that 69.8% and 37.6% of the respondents reacted with no importance of washing hands with soap after using a toilet/latrine and no need to treat or boil water before drinking, respectively.

**Conclusions:**

*A. lumbricoides* infection has a high prevalence in infants of the Kibaha District of Tanzania, especially in Kongowe village. The infection is dominant in males compared to female children. The prevalence is due to risk factors such as inadequate sanitation, low level of education, knowledge, and awareness of prevention strategies.

## 1. Introduction

Infections by soil-transmitted helminths are common in poor and destitute communities [1, 2]. The prone species that affect people are roundworms, whipworms, and hookworms [3]. *Ascaris lumbricoides* (roundworm) can grow up to a length of 35 cm and is associated with the helminthic disease, ascariasis. *A. lumbricoides* infect man after an ingested fertilised cyst develops into a larval worm and infiltrates the walls of the duodenum into the bloodstream. Through the bloodstream, it penetrates the pulmonary circulatory system, liver, and heart where it enters into the alveoli to moult and grow [1].

Inside the intestines of the host, *A. lumbricoides* produces thousands of eggs each day The cysts (eggs) are passed in the focal excrete of infested persons during egestion; later, these eggs are transmitted to another host by ingestion; thus, this cycle is easily adopted where there is inadequate sanitation [4]. *A. lumbricoides* cysts can be ingested by infants while playing with soil as they put their hands in their mouths without washing and also by drinking untreated water from contaminated source [5]. In some situations, the cysts that are attached to vegetables are ingested when these vegetables are not well pecked, washed, or cooked [6].

The worldwide distribution and occurrence show that more than 1.8 billion people who account for 28% of the global population are infected with *A. lumbricoides* infection [2]. *A. lumbricoides* infection is extensively distributed in East Asia and sub-Saharan Africa [7]. According to Lewetegn et al., over 835 million children living in East Asia and sub-Saharan Africa necessitate preventive measures and treatment against the *A. lumbricoides* parasitic worm [8]. The worldwide target is to eradicate illness caused by *A. lumbricoides* in infants by 2030; this will be achieved by frequently treating at least 85% of the children in prevalent areas, and this is estimated to be 980 million in the year 2027 [9].

*A. lumbricoides* weaken the nutritional status of the affected personnel in various ways, and this includes feeding on the host tissue like blood, which leads to malabsorption of nutrients like protein and loss of iron [10]. Additionally, roundworms compete for vitamin A in the host intestine. *A. lumbricoides* has been associated with loss of appetite, leading to a decrease in food intake and thus affecting the physical aptness of a person; moreover, it is known to cause diarrhoea and dysentery [1].

After several years of *A. lumbricoides* control, still, the infection is high in Tanzania as it ranges between 22% in the urban and 70% in rural [5]. In 2019, the World Health Assembly unanimously recognized a determination that urged prevalent nations, including Tanzania, to tackle worms, precisely *A. lumbricoides*, by providing surveillance of the extensiveness of the infection and deworming [11]. The World Health Organization (WHO) recommends deworming to be given annually when the baseline frequency of *A. lumbricoides* infection is found to affect below 50% of the infant population of a given community and two times a year when the occurrence is found to affect over 50% of the community [3]. Survey of the prevalence of worm infestation and treatment reduces morbidity by dropping the burden of the worm; additionally, health and sanitation education reduces spread and reinfection by encouraging health behaviours [6].

Despite the Pwani Region being highly populated, it has been recorded to be affected by several hygienic risk factors such as lack of latrines, poor sewage waste disposal, and insufficient clean drinking water [12, 13]. *A. lumbricoides* is a common parasite among infants; still, there is less information concerning its prevalence of infection among children under the age of five years old in Tanzania [5]. This research tends to explore more about the status of *A. lumbricoides* infection and provide data for governmental and nongovernmental institutions in the elimination of *A. lumbricoides* in Kibaha District located in Pwani Region, Tanzania.

## 2. Materials and Methods

### 2.1. Study Area

The investigation was carried out at Kongowe Health Centre at Kongowe village, Kibaha District of Pwani Region, Tanzania (6° 47′ 59^″^ S, 38° 53′ 59^″^ E). According to the household population census done in Tanzania in the year 2022, Kibaha District has a population of 161,925 people and 38,807 of the total population are children aged 0-5 years old [14]. Being one of the active government health centres in Kibaha District, it receives a recommendable number of child patients due to its well-known pediatric services that are appreciated by the community.

### 2.2. Study Design and Population

A cross-section of research was done on 400 patients; this included 170 and 230 males and females, respectively, who were below the age of 5 years old at both the outpatient and inpatient departments at Kongowe Health Centre from October 2022 to January 2023. The sampling was equally done on both the male and female genders. The sample size of the population in this study was determined with the formulation adopted by Harizanov et al. [2]:

(1) $$\begin{array}{c} N=\frac{Z^{2}P\left(1-P\right)}{E^{2}}, \end{array}$$

where *N* is the desired sample size, *Z* = 1 : 96 (standard normal deviation at 95% confidence interval), *P* is the estimated prevalence of *A. lumbricoides* infection to be 10.1%, and *W* is the margin of error to be 5%.

Patients within the age group of 0 to 5 years visiting the pediatric and laboratory departments during the data collection period and their parents willing to let them participate in this study were included. Children who visited the hospital and had been dewormed within one month before the study and those whose parents and guardians refused to be questioned were not included in the study.

### 2.3. Laboratory Testing Using Human *A. lumbricoides* IgG ELISA Kit and Colourimetry Technique

Determination of the prevalence of *A. lumbricoides* was done by laboratory testing of the infants using human *Ascaris lumbricoides* immunoglobulin G (enzyme-linked immunosorbent assay) kit (human *A. lumbricoides* IgG ELISA kit) and a colourimetry technique. Human *A. lumbricoides* IgG ELISA kit is composed of *A. Lumbricoides*-coated microplate (IgG), sample diluent, stop solution, washing buffer (20× conc.), horseradish peroxidase- (HRP-) labelled protein A conjugate, 3,3′,5,5′-tetramethylbenzidine (TMB) substrate solution, *A. lumbricoides* IgG positive control, *A. lumbricoides* IgG cut-off control, and *A. lumbricoides* IgG negative control. The human *A. lumbricoides* IgG ELISA kit was considered to determine in vitro levels of human IgG class antibodies to *A. lumbricoides* (*A. lumbricoides* IgG) in either plasma or serum samples since its analytical sensitivity is below 95% [11].

The assay involved obtaining serum from the blood of the patient using the centrifugation technique. Using a 1000 *μ*L micropipette, add 100 *μ*L of the patient's serum and negative and positive controls into the 96-well plate precoated with *A. lumbricoides* antigen and incubate at 37°C for 1 hour to bind with the cognate antibodies. Wash each well three to four times with 320 *μ*L of 20× washing buffer solution after incubation. The immersion time between each wash cycle should be >5 seconds. Decant the remaining washing buffer after performing the last wash. Remove the extra liquid by flip-flopping the plate and blotting on clean filter paper. To the wells of immobilized *A. lumbricoides*-specific antibodies, add 110 *μ*L horseradish peroxidase- (HRP-) labelled protein A conjugate before incubation at 37°C for 30 minutes. To the wells, add 100 *μ*L of TMB and incubate for 15 minutes at 37°C. After incubation, add the stop solution and determine the density by measuring the absorbance of the specimen at 450 nm. The concentration of yellow colouration is directly proportional to the amount of *A. lumbricoides* IgG samples captured in the plate. The recorded results were reviewed along with the child's parents to confirm participation in the research and documented accordingly.

### 2.4. Factors Related to *A. lumbricoides* Infection

Information concerning behavioural factors, sociodemographic and biochemical parameters, economic factors, and physical measurements was collected from the parents of the children who were tested for *A. lumbricoides* infection using pretested questionnaires with close-ended questions.

### 2.5. Data Analysis

Data for *A. lumbricoides* infection measured were subjected to analysis by Microsoft Excel and then displayed as graphs, while data for sociodemographic and risk assessment findings were subjected to bivariate and multivariable analysis by Statistical Package for Social Sciences version 29 software (IBM SPSS Inc., USA) at a level of significance of *P* < 0.05.

### 2.6. Ethical Consideration

This research study was permitted by the Department of Microbiology and Parasitology before approval by the Health Ethical Committee, Faculty of Medicine and Pharmaceutical Sciences at Kampala International University in Tanzania, no. 143/FMPS/MPI/2022, on the 20^th^ of April 2022.

## 3. Result

### 3.1. Sex and *A. lumbricoides* Infection

**Figure 1:**
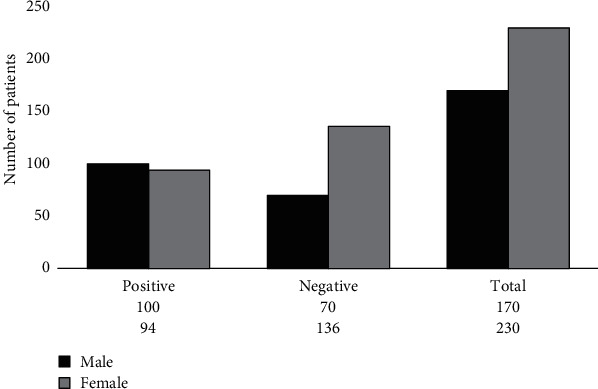
Gender and status of *A. lumbricoides* infection in the children who participated in the research.

Out of the 400 children (170 males and 230 females) who participated in the laboratory testing of *A. lumbricoides* infection, 59% (100/170) of the tested males and 41% (94/230) of the tested females were found to be positive (Figure 1).

### 3.2. Age and *A. lumbricoides* Infection

**Figure 2:**
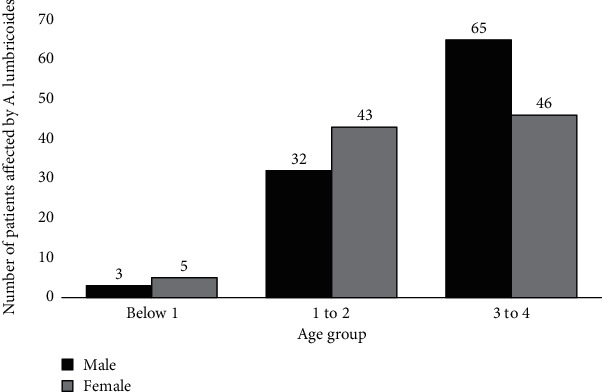
The distribution of *A. lumbricoides* infection among the age groups of infants who were found positive.

Amongst the 194 children who were found to be positive (100 males and 94 females), 4.1%, 38.7%, and 57.2% were found to be in the age group of below 1, 1 to 2, and 3 to 4, respectively (Figure 2).

### 3.3. Sociodemographic Findings and the Risk Factors Related to *A. lumbricoides* Infection

Out of the 400 questionnaires given to the parents of children who underwent laboratory tests for *A. lumbricoides* infection, 320 questionnaires were answered and returned. The tabulated responses showed that 262 (81.8%) of the respondents at *P* = 0.041 and 258 (80.6%) at *P* = 0.101 knew that *A. lumbricoides* infection is acquired from faeces and uncooked food, respectively. From the questionnaires, 198 (61.8%) at *P* = 0.132 and 202 (63.2%) at *P* = 0.085 respondents did not accept that soil and dirty water, respectively, were not sources of *A. lumbricoides* infection (Table 1). At *P* = 0.612 and *P* = 0.022, 64% and 91.2% of the respondents showed the importance of using a latrine/toilet and washing fruits, respectively, as 69.8% and 37.6% showed no importance of washing hands with soap after using a toilet/latrine and no need of treating or boiling water before drinking, respectively (Table 1).

## 4. Discussion

Many studies have investigated the contribution of *A. lumbricoides* infection to child growth and malnutrition despite the difficulties in knowing its prevalence and dominance, especially in sub-Saharan African countries including Tanzania. Kibaha District is one of the populated areas in the Pwani Region of Tanzania [14]; this study revealed that there is dominance of *A. lumbricoides* infection in children under the age of five years old in Kibaha District, and results show that 194 out of the 400 children who underwent laboratory diagnostics at Kibaha Health Centre were found to harbour *A. lumbricoides.*

Harizanov et al. and Brooker and Bundy stated that *A. lumbricoides* infection is one of the world's most prevalent afflictions of humans who live in an area of poverty and densely populated zones in tropical and subtropical countries [2, 7]. Similar studies done in Temeke District and Kiwangwa rural ward, Bagamoyo District, Tanzania, revealed that 200 children out of 300 tested children aged 6 years old and below who were tested in a survey that took 3 months in the year 2021 had *A. lumbricoides* infection [15–17]. This study reported that *A. lumbricoides* infection is high in males (100) compared to female (94) infants; this was comparable to a study done by Makata et al. at Bukoba urban, Bukoba rural, and Muleba in the Kagera Region which reported that out of 250 infants, 100 males and 30 females were infected with *A.* lumbricoides [18]. Other similar studies include studies by Nvule, Kabatende et al., and Inocencio et al., where they displayed higher infections in male than female children at the Pediatric Unit of Kiwoko Hospital, Nakaseke District of Uganda, Western Province of Rwanda and Kwilu Province in the Democratic Republic of the Congo, respectively [19–21]. Contrary, Bogoch et al. and Sartorius et al. reported a high number of infections in girls as in boys aged 0 to 3 years old [22, 23].

This study showed that infants aged between 3 to 4 years old are highly affected by *A. lumbricoides* compared to kids aged 0 to 2 years old. Studies in Temeke District and Pemba Island showed that kids aged 2 to 4 years were highly affected by *A. lumbricoides* [16, 22]. These results might be due to the active and playful nature of infants as they grow up. Most kids play with toys, dirt, and soil in their surroundings, and more so, some tend to lick their hand fingers with dirt and put toys in their mouth during their playing time. These dirty toys and contaminated soil always harbour the eggs of *A. lumbricoides* [5].

Control of *A. lumbricoides* infection has always depended on medications like albendazole and mebendazole [6]. This research is aimed at determining the prevalence and getting to know the public knowledge on preventive measures that can be implored. Statistical analysis showed that *A. lumbricoides* infection is acquired from faeces and the respondents showed the importance of using latrines/toilets. This was similar to the report by Makata et al.; in their survey of northwestern Tanzania, they reported that latrines were highly demanded by the residents of Bukoba and Muleba to prevent contamination of soil by improper disposal of faeces [18]. Research by Imalele et al. recorded that out of 280 respondents, 95% suggested that the use of bushes instead of toilets was the cause of contamination of the soil by *A. lumbricoides* [24]. Toilets and latrines are major areas for the disposal of faeces; proper disposal of faeces and sewage effluent is vital in the prevention of the spread of water-borne diseases and parasites including *A. lumbricoides* to the environment [12].

This study recorded that eating uncooked foods and not washing fruits before eating played an essential role in *A. lumbricoides* infection. Several reports support this record. The World Health Organization recommends the proper cooking of foods like meat and washing of fruits to remove dirt before consumption [8]. Out of 100 participants in a study conducted in Central Java, Indonesia, 72% of the participants acknowledged that food needs to be cooked properly to prevent the spread of tapeworms and *Ascaris* [9]; a similar report was displayed by Al-Tameemi and Kabakli in Andhra Pradesh, India, where they reported that eating raw food without proper cooking and using dirty water to wash fruits and vegetables are part of the *A. lumbricoides* infection [6].

This research was contrary to data conveyed by Kabatende et al. in Rwanda and Lewetegn et al. in North Shoa, Ethiopia, where they reported that soil and dirty water contaminated with sewage effluents were sources of *A. lumbricoides* infection [8, 20]. Various research parasitologists have reported that eggs of *A. lumbricoides* are normally acquired in soil by children when playing since they tend to put their hands in their mouths as they play; more so, contaminated water with sewage contains the eggs of the *A. lumbricoides* and taken in by orally during drinking of water [5, 12].

Records from Bagamoyo District in Tanzania and Nakaseke District in Uganda show that washing hands using soap after visiting the toilet is mandatory as a measure of preventing contamination by *A. lumbricoides* infection [15, 19, 25]. Further results by Lee et al. and Mwakitalima et al. displayed that it is essential to chemically treat water or boil it before drinking [9, 12]. According to WHO, washing hands helps to remove the cysts that stick on the hands after visiting the toilet, and also eggs that might have been acquired while touching dirty surfaces [5]; furthermore, boiling drinking water kills the eggs of *A. lumbricoides*, microorganisms, and other parasitic worms found in the water ecosystem, thus being safe for drinking [3, 26].

This study has shown that most participants were affected with *A. lumbricoides* infection because of risk factors associated with poor knowledge as indicated in the survey by Harizanov et al. who reported that people who are infected with these parasites occur most frequently in developing tropical countries, due to risk factors such as poverty, low level of education and awareness, poor health services, inadequate sanitation, and knowledge on how to prevent these infections [2].

## 5. Conclusion

*A. lumbricoides* infection has a high prevalence in infants of the Kibaha District of Tanzania, especially in Kongowe village. The infection is dominant in males as compared to female children. The prevalence is due to risk factors such as inadequate sanitation, low level of education, knowledge, and awareness of prevention strategies.

## 6. Recommendations

This study has shown that *A. lumbricoides* infection is a threat in Kibaha District of Tanzania; thus, proper mitigation and recommendable strategies like hygiene practices are essential in the eradication of this parasitic worm especially in infants aged 0 to 5 years of age. More so, awareness and educating the residents of Kongowe village concerning *A. lumbricoides* and other parasitic worms will be of great importance. Furthermore, periodic hospital checkups of infants to know their health status will aid in knowing the status of infants in Kongowe village concerning *A. lumbricoides*. An extensive study on the prevalence of *A. lumbricoides* in the whole of the whole region of Pwani is highly recommendable.

## 7. Limitations of the Study

This research was limited by duration and economic constraints to analyze a large sample size.

## Figures and Tables

**Table 1:** Bivariate and multivariable analysis of risk factors associated with *A. lumbricoides* infection.

	Response	Frequency number of responses (%)	*P* value	Odds ratio
Source of *A. lumbricoides* infection				
Faeces	Yes	262 (81.8%)		
	No	44 (13.9%)	0.041	0.283
	Do not know	14 (4.3%)		
Soil	Yes	50 (15.6%)		
	No	198 (61.8%)	0.132	0.661
	Do not know	72 (22.6%)		
Dirty water	Yes	73 (22.7%)		
	No	202 (63.2%)	0.085	0.164
	Do not know	43 (14.1%)		
Uncooked food	Yes	258 (80.6%)		
	No	45 (14.1%)	0.101	0.122
	Do not know	17 (5.3%)		
Prevention of *A. lumbricoides* infection				
Use of latrines/toilets	Use of latrines/toilets	241 (75.4%)		
	No need for latrines/toilets	64 (20.1%)	0.612	0.321
	Any place (latrine, bush) as long as to defecate	15 (4.5%)		
Wash your hands with soap after using the toilet/latrine	Wash with soap	125 (39.2%)	0.155	0.044
	No wash with soap	195 (69.8%)		
Treatment of water for drinking	Boiling water	97 (30.4%)		
	Water guard	103 (32.0%)	0.394	0.049
	No treatment	120 (37.6%)		
Wash fruit before eating	Wash fruit	292 (91.2%)	0.022	0.307
	No wash of fruit	28 (8.8%)		

## Data Availability

The data used to support the findings of this study are available from the corresponding author upon request.
